# The association between sedentary behavior and obstructive sleep apnea: a cross-sectional study from the NHANES (2007–2008 to 2015–2020)

**DOI:** 10.1186/s12903-024-03960-0

**Published:** 2024-02-12

**Authors:** Song Cai, SiYu Li, YaShi Zhou, JuKun Song, JuXiang Peng

**Affiliations:** 1 School of Stomatology, Zunyi Medical University, Zunyi, Guizhou China; 2 Guiyang Stomatological Hospital affiliated to Zunyi Medical University, Guiyang, Guizhou China; 3 Department of Oral and Maxillofacial Surgery, The Affiliated Stomatological Hospital of Guizhou Medical University, Guiyang, Guizhou China; 4 School of Stomatology, Zunyi Medical University, Zunyi, Guizhou China; 5 Guiyang Hospital of Stomatology, Guiyang, Guizhou China

**Keywords:** Sedentary behavior(SB), Obstructive sleep apnea(OSA), NHANES

## Abstract

**Background:**

Sedentary behavior (SB) may contribute to obesity and lower extremity fluid retention, which may favor the development of obstructive sleep apnea (OSA). However, linking sedentary behavior to OSA is unclear. The purpose of this study was to determine if there is an association between SB and OSA.

**Methods:**

Three typical questions in the NHANES questionnaire(①The frequency of feeling excessively sleepy per month. ②The frequency of gasping, snorting or stopping breathing per week. ③The frequency of snoring per week.) have been used for the assessment of OSA. A physical activity questionnaire(On a typical day, the amount of time you spend sitting or reclining.) was used to assess SB. This secondary analysis included National Health and Nutrition Examination Survey (NHANES) participants (unweighted = 20,115). Weighted sample and multiple logistic regression complex sample analysis techniques were used in this study.

**Results:**

After adjustment for confounders, participants with SB(> 8 h/d) had a higher risk of OSA compared to SB(< 4 h/d). Stratified analysis by gender showed that there was no significant association of SB and OSA in men. However, in women, with SB(< 4 h/d) as the reference, participants with(≥ 4 h/d) had an increased risk of OSA. By age-stratified analysis, the association of SB with OSA was stronger among older participants.

**Conclusion:**

Analysis in this study showed a positive association between SB and OSA, more pronounced in women and participants older than 60 years old.

**Supplementary Information:**

The online version contains supplementary material available at 10.1186/s12903-024-03960-0.

## Introduction

Obstructive sleep apnea (OSA) is a condition in which the upper airway repeatedly collapses or completely blocks during sleep. The manifestations of OSA are characterized by a lack of specificity and include snoring, arousals, apneas, and excessive daytime sleepiness [1]. Previous studies have highlighted that OSA usually occurs in the context of multiple comorbidities, including obesity, high blood pressure(HBP), dyslipidemia, diabetes, etc [2]. In addition, OSA is also related to psychological stress [3]. Worldwide, 1 billion people aged 30 to 69 years old maybe diagnosed the disease in the world, according to Benjafield AV et al [4]. Approximately 425 million people are believed to have moderate to severe OSA, which is generally recommended for treatment [4]. OSA is generally not well understood in developing countries, diagnostic and therapeutic modalities are unavailable or not modified for resource-limited settings [5]. Approximately $12.4 billion was spent in the United States in 2015 to diagnose and treat OSA [6]. Studies have shown that the prevalence of OSA has exceeded 50% in Germany, Japan, and Poland [7].The high prevalence of OSA in China, despite a low prevalence of obesity, suggests that craniofacial and other phenotypic abnormalities may contribute to this phenomenon [8].

Significant neurocognitive and cardiovascular sequelae are also associated with OSA [9]. Sedentary behavior (SB), defined as reclining or sitting and low energy activities such as computer use, driving, screen time and reading [10], plays a major role in developing Cardiovascular disease(CVD) [11]. Previous epidemiologic studies have shown conflicting results regarding the association between SB and OSA, with some studies suggesting a positive association [12–14], others finding no association. Saurabh S Thosar et al. argued that shorter sleep was predictive of more time spent sitting during the next day in healthy middle-aged adults, but not in sleep apnea patients [15]. However, previous studies had a relatively small sample sizes or inadequate adjustment for important confounders (Physical Activity(PA), Body Mass Index(BMI), Sleep Duration etc.). In addition, as far as we know, there is little evidence to suggest an association. Therefore, in this study, the purpose was to investigate the association of SB with OSA by large, representative population from NHANES.

## Materials and methods

### Data source

This cross-sectional research employs the publicly accessible NHANES dataset, without the necessity for further ethical review committee approvals (https://wwwn.cdc.gov/nchs/nhanes/Default.aspx).

### Population of the study

The data analyzed in this study were designed to assess the health and nutritional status of adults and children in the United States from the National Health and Nutrition Examination Survey (NHANES). Since 1999, data have been collected in 2-year cycles by the National Center for Health Statistics (NCHS). The NHANES information collection and methodology have been detailedly elaborated on the website(http://www.cdc.gov/nchs/nhanes.htm), which is accessible [16].

**Fig. 1:**
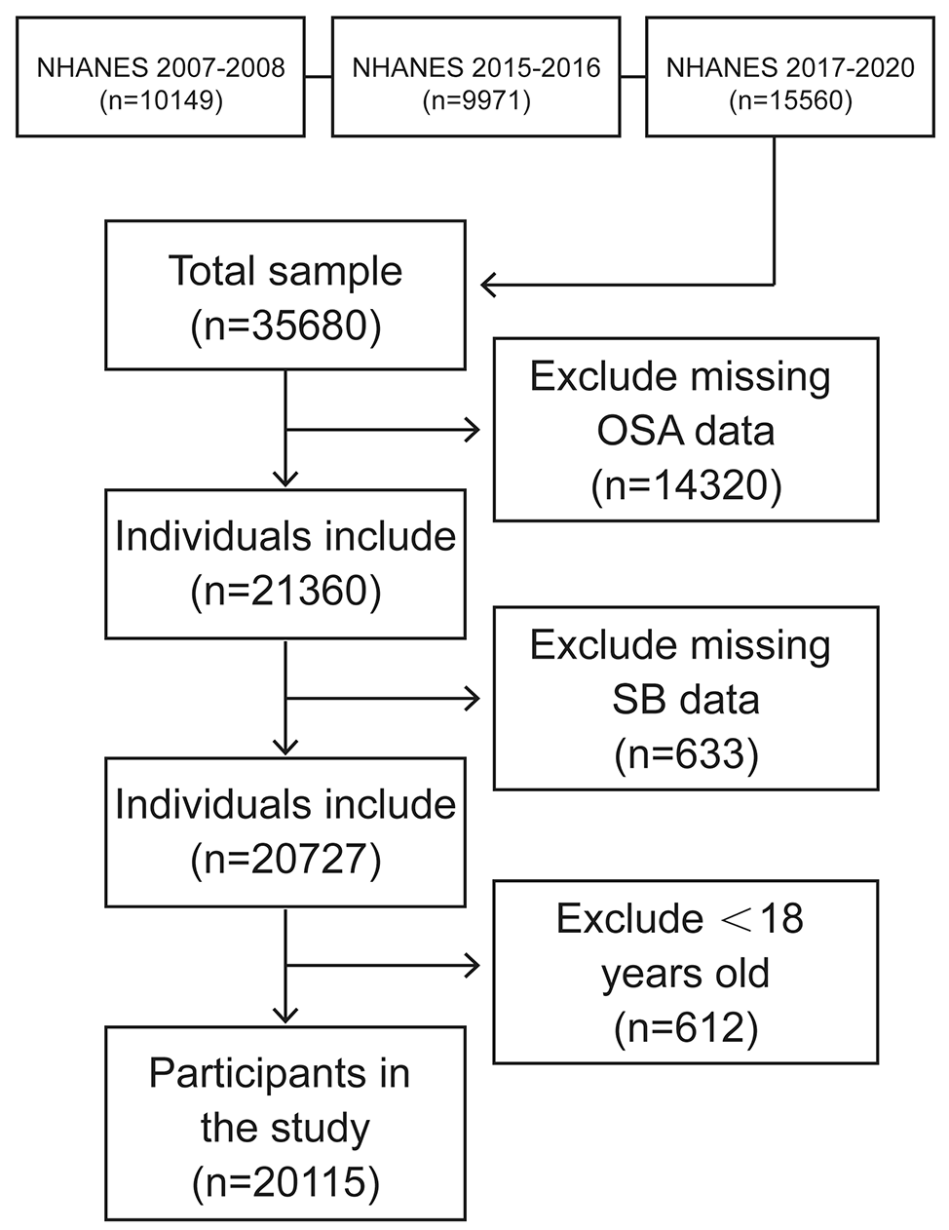
Flow chart of the study design and participants excluded from the study

Data from 4 cycles (2007–2008、2015–2020) with a total of 35,680 subjects were analyzed. There were 14,320 participants with missing OSA data that were excluded in this study. Additionally, 633 participants were excluded. These participants had incomplete data on SB. Further, We excluded participants younger than 18 years old(*n* = 612), The final sample analyzed in this study was 20,115, all of whom were 18 years of age or older. The flow chart for population screening is as follows. (Fig. 1).

### Obstructive sleep apnea (OSA)

Obstructive sleep apnea (OSA), according to previous report, was defined as participants answering “yes” to at least one of the following three NHANES dichotomous questions [17]: ①Feeling excessively sleepy during the day 16–30 times per month despite sleeping approximately 7 or more hours per night on weekdays or work nights; ②Gasping, snorting or stopping breathing 3 or more nights per week; ③Snoring 3 or more nights per week.

### Sedentary behavior (SB)

The assessment of sedentary behavior in NHANES was a self-report question. The question the participants heard was that how much time do you usually spend sitting or reclining on a typical day? Specifically, in 2007–2008 survey cycle, the above question only included sitting time per day. For the 2015–2016, 2017–2020 survey cycles, the above question included daily sitting and reclining time. Behaviors that accompany SB include: getting to and from places, at home, sitting at school, or with friends, including time spent sitting at a desk, reading, traveling in a car or bus, playing cards, watching television, or using a computer. It doesn’t account for time spent sleeping. Self-reported sitting or reclining time was categorized as four levels of < 4 h/d, 4 to < 6 h/d, 6 to 8 h/d, and > 8 h/d [18].

### Assessment of other covariates

On the basis of clinical judgment and previous research [16], the following covariates were included: age (year), sex (male/female), race/ethnicity (non-Hispanic black, Mexican American, non-Hispanic white, other race/ethnicity), education level (less than high school, high school, college or more), marital status (never married, married/cohabiting, widowed/separated/divorced), poverty income ratio (PIR). BMI (the measured weight in kilograms is divided by the measured height in meters squared) was divided into four groups (< 25.0, 25.0-<30.0, 30.0-<40.0, ≥ 40.0), and BMI ≥ 40 kg/m^2^ was considered morbid obesity [19, 20]. Smoking status was categorized as never smoker (smoked < 100 cigarettes in lifetime), former smoker (smoked > 100 cigarettes but currently quit smoking), current smoker (smoked > 100 cigarettes and currently smoking) [21]. According to past research [22], we defined moderate drinking as 14 or fewer drinks/week for men or 7 or fewer drinks/week for women or 5 or fewer drinks/day on any single day in the past year for bothr men and women. Similarily, we defined heavy drinking as more than 14 drinks/week for men or more than 7 drinks/week for women or 5 or more drinks/day on any single day at least once in the past year for either men or women [22]. Physical activity was categorized as vigorous/moderate activity (more than 150 min/week of moderate-intensity aerobic activity or 75 min/week of vigorous-intensity aerobic activity or an equivalent combination of moderate and vigorous activity [23]. Sleep duration was categorized as inadequate(<7 h/d), recommended(7-9 h/d), excessive sleep duration(>9 h/d) [22]. High blood pressure(HBP) and diabetes are also included in the study as covariates. Based on recent research [24], HBP was either determined by blood pressure measured in NHANES (≥ 130 mm Hg (systolic) or ≥ 80 mm Hg (diastolic)) or self-reported by participants who were diagnosed by a healthcare professional. A history of diabetes was self-reported by participants who were diagnosed by a healthcare professional or determined by reviewing prescriptions for medications used to treat diabetes.

### Statistical analysis

Both descriptive and regression analyses using weighted samples were conducted in this study. Descriptive analysis expressed categorical variables as weighted percentages (%) with 95% confidence intervals. To calculate the percentages of categorical variables between the groups with and without OSA, weighted chi-squared tests were performed. Odds ratios (ORs) and 95% confidence intervals (CIs) were calculated for associations between sedentary behavior (SB) and obstructive sleep apnea (OSA) by constructing three multivariable logistic regression models. In model I, no covariates were adjusted. In model II, age, sex, and race/ethnicity were adjusted. In model III, age, sex, race/ethnicity, HBP, diabetes, PA, smoking, drinking, sleep duration, BMI, PIR, education level, and marital status were adjusted. Sedentary behavior was categorized into four levels, and the lowest level was used as the reference. Subgroup analyses stratified by sex and age were also performed. We conducted all analyses in Stata version 16.0 and Empowerstats version 2.0. To account for the sampling design, sample interview weights were applied. *P* value<0.05 is statistically significant.

## Result

### Participants’ baseline characteristics

Overall, 20,115 participants were eligible for final analysis, and the weighted population was 165,457,386. The individual characteristics of the subjects with and without OSA are shown in Table 1. Of the 20,115 participants analyzed, 10,651 individuals were included in the OSA group and the weighted prevalence was 52.95%. 42.30% of the participants were younger than 44 years and 50.75% were female. In both OSA and non-OSA groups, sex, age, race, education level, marital status, PIR, alcohol consumption, BMI, high blood pressure(HBP), diabetes, PA, SB, sleep duration and smoking were significantly different. Those diagnosed with OSA, according to Table 1, were more likely to be male, be non-hispanic white, have a college degree or higher, be married or living with a partner, have obesity, have a diagnosis of HBP or diabetes, engage in moderate to vigorous physical activity, sleep for 7–9 h per day, and be nonsmokers. Among all populations with OSA, men and women differ in BMI, history of diabetes, drinking habits, marital status, physical activity, poverty income ratio(PIR), race, sleep duration, and smoking habits (Table S1).

**Table 1:** Baseline Participant Characteristics(*N* = 20,115)

		Obstructive Sleep Apnea(OSA)		
Characteristics	Total(*N* = 20,115)	No (*N* = 9,464)	Yes(*N* = 10,651)	*P*-value
**Sex**				< 0.001
Male	9906 (49.25%)	4241 (44.81%)	5665 (53.19%)	
Female	10,209 (50.75%)	5223 (55.19%)	4986 (46.81%)	
**Age**				< 0.001
<44	8508 (42.30%)	4743 (50.12%)	3765 (35.35%)	
[44,60)	5059 (25.15%)	1974 (20.86%)	3085 (28.96%)	
≥ 60	6548 (32.55%)	2747 (29.02%)	3801 (35.69%)	
**Race/Ethnicity**				0.007
Non–Hispanic white	7442 (37.00%)	3515 (37.14%)	3927 (36.87%)	
Non–Hispanic black	4676 (23.25%)	2226 (23.52%)	2450 (23.00%)	
Mexican American	3003 (14.93%)	1327 (14.02%)	1676 (15.74%)	
Other Race/Ethnicity	4994 (24.83%)	2396 (25.32%)	2598 (24.39%)	
**Education Level**				< 0.001
Below high school	4435 (22.05%)	1846 (19.51%)	2589 (24.31%)	
High school	4487 (22.31%)	1975 (20.87%)	2512 (23.59%)	
College or above	10,202 (50.72%)	4965 (52.46%)	5237 (49.17%)	
**Marital Status**				< 0.001
Never married	3453 (17.17%)	1958 (20.69%)	1495 (14.04%)	
Married/Living with partner	11,671 (58.02%)	5062 (53.49%)	6609 (62.05%)	
Widowed/Divorced/Separated	4003 (19.90%)	1768 (18.68%)	2235 (20.98%)	
**PIR**				0.009
<1	3687 (18.33%)	1768 (18.68%)	1919 (18.02%)	
[1, 3)	7641 (37.99%)	3480 (36.77%)	4161 (39.07%)	
≥ 3	6386 (31.75%)	3052 (32.25%)	3334 (31.30%)	
**Alcohol Drinkers**				< 0.001
Non-drinkers	2403 (11.95%)	1295 (13.68%)	1108 (10.40%)	
Moderate alcohol use	7670 (38.13%)	3507 (37.06%)	4163 (39.09%)	
Heavy alcohol use	7220 (35.89%)	3186 (33.66%)	4034 (37.87%)	
**BMI**				< 0.001
<25	5282 (26.26%)	3352 (35.42%)	1930 (18.12%)	
[25,30)	6022 (29.94%)	2853 (30.15%)	3169 (29.75%)	
[30,40)	5880 (29.23%)	2134 (22.55%)	3746 (35.17%)	
≥ 40	1520 (7.56%)	402 (4.25%)	1118 (10.50%)	
**HBP**				< 0.001
No	8233 (40.93%)	4575 (48.34%)	3658 (34.34%)	
Yes	10,312 (51.27%)	3991 (42.17%)	6321 (59.35%)	
**Diabetes**				< 0.001
No	1425 (7.08%)	566 (5.98%)	859 (8.07%)	
Yes	2907 (14.45%)	989 (10.45%)	1918 (18.01%)	
**PA**				< 0.001
Light	4083 (20.30%)	2058 (21.75%)	2025 (19.01%)	
Moderate to vigorous	5211 (25.91%)	2744 (28.99%)	2467 (23.16%)	
**SB**				< 0.001
<4 h/d	6122 (30.44%)	2984 (31.53%)	3138 (29.46%)	
4 to<6 h/d	5039 (25.05%)	2395 (25.31%)	2644 (24.82%)	
6 to 8 h/d	5320 (26.45%)	2494 (26.35%)	2826 (26.53%)	
>8 h/d	3634 (18.07%)	1591 (16.81%)	2043 (19.18%)	
**Sleep duration**				< 0.001
<7 h	5626 (27.97%)	2249 (23.76%)	3377 (31.71%)	
[7,9]	12,409 (61.69%)	6158 (65.07%)	6251 (58.69%)	
>9 h	1987 (9.88%)	1020 (10.78%)	967 (9.08%)	
**Smokers**				< 0.001
Never smoker	11,533 (57.34%)	5933 (62.69%)	5600 (52.58%)	
Former smoker	4552 (22.63%)	1816 (19.19%)	2736 (25.69%)	
Current smoker	3738 (18.58%)	1527 (16.14%)	2211 (20.76%)	

Values are expressed as N(%) for categorical variables. P-value was calculated using the weighted chi-squared test for categorical variables

PIR = Poverty In Ratio; BMI = Body Mass Index; HBP = High Blood Pressure; SB = Sedentary Behavior; PA = Physical Activity

Numbers that do not add up to 100% are attributable to missing data

### Effects of SB on OSA

Three sets of weighted logistic regression models were constructed. The relationship between sedentary behavior and OSA is shown in Table 2(Table 2). In model I, with SB < 4 h/d as a reference, participants with SB 6 to 8 h/d had a high risk of OSA(OR = 1.08,95%CI(1.00-1.16),*P* = 0.047), and participants with SB > 8 h/d had a higher risk of OSA(OR = 1.22,95%CI(1.12–1.33),*p*<0.001). However, this association was not significant in participants with SB 4 to <6 h/d(OR = 1.05,95%CI(0.97–1.13),*P* = 0.202). Furthermore, the trends remained the same after adjustment for age, sex, and race (model II). When further adjusted for education level, physical activity, smoking status, BMI, PIR, HBP, diabetes, drinking status, marital status, and sleep duration (model III), the OR for participants with SB > 8 h/d was 1.22 and 95%CI(1.12–1.34)(*P*<0.001). Prolonged sedentary behavior had a positive association with OSA.

**Table 2:** The association between Sedentary Behavior and OSA

	Model I OR(95%CI)p-value	Model II OR(95%CI)p-value	Model III OR(95%CI)p-value
Sedentary Behavior(hours/day)			
0 to < 4	reference	reference	reference
4 to < 6	1.05 (0.97, 1.13) 0.202	1.06 (0.99, 1.15) 0.112	1.07 (0.99, 1.16) 0.097
6 to 8	1.08 (1.00, 1.16) 0.047	1.09 (1.01, 1.18) 0.019	1.08 (0.99, 1.16) 0.077
> 8	1.22 (1.12, 1.33) < 0.001	1.27 (1.17, 1.38) < 0.001	1.22 (1.12, 1.34) < 0.001
*P* for trend	< 0.001	< 0.001	< 0.001

OR, Odds Ratio; CI, Confidence Intervals; OSA, Obstructive Sleep Apnea.

We also analyzed the associations of sedentary behavior with OSA by gender(Table 3). However, the association was not significant in men(*P*>0.05) when stratified by gender. Consistent with the overall analysis, among women, with SB <4 h/d as the reference, participants with SB 4 to <6 h/d had a high risk of OSA(OR = 1.17,95%CI(1.05, 1.32),*p* = 0.005), participants with 6 to 8 h/d(OR = 1.12,95%CI(1.00, 1.25),*p* = 0.054), and participants with SB>8 h/d(OR = 1.35,95%CI(1.19, 1.54),*p*<0.001) in model III. Association of sedentary behavior with OSA varied by gender. Among those who were sedentary for ≥ 4 h/d, female gender was significantly associated with higher OSA risk. Sitting or reclining for ≥ 4 h/d is a risk factor for OSA in women.

**Table 3:** The association between Sedentary Behavior and OSA by gender

	Model I OR(95%CI)p-value	Model II OR(95%CI)p-value	Model III OR(95%CI)p-value
Stratified by gender			
Men(*n* = 9906)			
SB 0 to < 4	reference	reference	reference
SB 4 to < 6	0.95 (0.85, 1.06) 0.352	0.96 (0.86, 1.07) 0.470	0.95 (0.85, 1.06) 0.370
SB 6 to 8	1.02 (0.92, 1.13) 0.741	1.03 (0.92, 1.15) 0.610	1.01 (0.90, 1.14) 0.842
SB > 8	1.10 (0.98, 1.24) 0.108	1.12 (0.99, 1.26) 0.068	1.08 (0.95, 1.23) 0.262
Women(*n* = 10,209)			
SB 0 to < 4	reference	reference	reference
SB 4 to < 6	1.15 (1.04, 1.28) 0.009	1.17 (1.05, 1.30) 0.005	1.17 (1.05, 1.32) 0.005
SB 6 to 8	1.14 (1.03, 1.26) 0.013	1.15 (1.04, 1.28) 0.009	1.12 (1.00, 1.25) 0.054
SB > 8	1.35 (1.20, 1.52) < 0.001	1.42 (1.26, 1.60) < 0.001	1.35 (1.19, 1.54) < 0.001

OR, Odds Ratio; CI, Confidence Intervals; OSA, Obstructive Sleep Apnea

The model was not adjusted for the stratification variable itself in the subgroup analysis by sex

The associations of sedentary behavior with OSA were also analyzed by age(Table 4). In people aged <44 years, after adjusting for all confounding factors, the risk of OSA was higher in participants with SB >8 h/d(OR = 1.22,95%CI(1.06, 1.41),*p* = 0.006). Similarly, among people aged 44 to <60years, the risk of OSA was higher in participants with SB > 8 h/d(OR = 1.26,95%CI(1.07, 1.49),*p* = 0.006) after adjusting for part of confounding factors. In people older than 60 years, after adjusting for all confounding factors, the risk of OSA was 1.22 times higher for SB 4 to < 6 h/d, 1.16 times higher for SB 6 to 8 h/d and 1.39 times higher for SB > 8 h/d, with sedentary participation < 4 h/d as the reference. In addition, for the larger grade group, only a shorter amount of sedentary behavior was needed to increase the risk for OSA.

**Table 4:** The association between Sedentary Behavior and OSA by age

	Model I OR(95%CI)p-value	Model II OR(95%CI)p-value	Model III OR(95%CI)p-value
Stratified by age			
Age<44(*n* = 8508)			
SB 0 to < 4	reference	reference	reference
SB 4 to < 6	0.91 (0.81, 1.02) 0.095	0.91 (0.81, 1.02) 0.103	0.95 (0.83, 1.07) 0.379
SB 6 to 8	0.96 (0.86, 1.08) 0.475	0.97 (0.87, 1.09) 0.666	1.04 (0.92, 1.18) 0.555
SB > 8	1.15 (1.01, 1.30) 0.031	1.18 (1.04, 1.34) 0.010	1.22 (1.06, 1.41) 0.006
Age[44,60)(*n* = 5059)			
SB 0 to < 4	reference	reference	reference
SB 4 to < 6	1.15 (0.99, 1.34) 0.068	1.17 (1.00, 1.36) 0.051	1.13 (0.96, 1.33) 0.151
SB 6 to 8	1.17 (1.01, 1.36) 0.037	1.20 (1.03, 1.39) 0.022	1.13 (0.96, 1.33) 0.139
SB > 8	1.22 (1.04, 1.44) 0.017	1.26 (1.07, 1.49) 0.006	1.19 (0.99, 1.43) 0.058
Age ≥ 60(*n* = 6548)			
SB 0 to < 4	reference	reference	reference
SB 4 to < 6	1.17 (1.02, 1.33) 0.021	1.25 (1.10, 1.44) 0.001	1.22 (1.06, 1.40) 0.005
SB 6 to 8	1.14 (1.00, 1.30) 0.048	1.26 (1.10, 1.43) <0.001	1.16 (1.02, 1.34) 0.030
SB > 8	1.35(1.16, 1.57) <0.001	1.51 (1.29, 1.77) <0.001	1.39 (1.18, 1.63) < 0.001

OR, Odds Ratio; CI, Confidence Intervals; OSA, Obstructive Sleep Apnea

The model was not adjusted for the stratification variable itself in the subgroup analysis by age

## Discussion

We aimed to investigate the association of sedentary behavior with OSA, and the results showed that sedentary behavior may be associated with OSA. Furthermore, after adjusting for important confounders, participants in the group with SB (> 8 h/d) had a 1.22 times higher risk of OSA than others with SB (< 4 h/d). This finding is in agreement with previous studies [12–14]. Although some studies have suggested that SB and OSA are not linked [25], we believe that the reason for this difference may be due to different screening tools. Interestingly, sitting for < 8 h/d has no significant effect on OSA, whereas sitting for > 8 h/d increases the risk of OSA over time, which was not observed in the previous study.

Rostral fluid shift plays critical role in OSA and central sleep apnea(CSA) progression [26]. When you lie in bed during the night, body fluid flows to the neck, increasing the resistance of the upper airway and causing the upper airway to collapse, exacerbating OSA [27]. In the population, the severity of OSA is related to posture in 50% of patients [28]. We followed the established definition of SB in this study [10] and in addition to contributing to obesity, excessive sitting during the day can lead to fluid retention in the lower body, especially in the legs [29]. we consider that the underlying mechanism of SB associated with OSA is that SB cause fluid accumulation in the lower limbs, which promotes the development of OSA during sleep due to rostral fluid shift. This finding aligns with previous research, which demonstrates that diminishing fluid accumulation is attainable through either a decrease in sedentary behavior or an enhancement in physical activity. Such changes have been shown to lower the risk of developing OSA [30, 31].

More importantly, there was another notable finding in our study. After stratifying by gender, we found that the association between sedentary behavior and OSA differed by gender. Being female was significantly associated with a higher risk of OSA. Again, we confirmed a prospective study with similar findings [14]. Our study supports that OSA is more common in men [32], while the results of this study also suggest that the association of sedentary behavior with OSA is more pronounced in women. With regard to the latter, the reason for this difference may be the higher estrogen levels in women and warrants further investigation. Examining the correlation between sedentary behavior (SB) and obstructive sleep apnea (OSA) across different age groups revealed an escalated association strength between SB and OSA with advancing age. Among older participants, even shorter daily durations of sedentary behavior amplified the risk of developing OSA. While previous research has demonstrated that OSA prevalence rises with age and BMI, further investigation is required to thoroughly explore the nuances of age-related disparities in the relationship between SB and OSA [33]. .

There are several strengths in our study. We used the large, representative NHANES population. In addition, the study adjusted for potential social demographic and lifestyle confounders to obtain more robust results. Furthermore, we use weighted sample analyses to examine the association. On this basis, the results were more generalizable. At the same time, several limitations should be noted when interpreting this article. First, we diagnose OSA on the basis of some typical symptoms found in NHANES questionnaire, such as daytime sleepiness, apnea, snoring, etc. And recall or self-report bias becomes a limitation with the use of questionnaires to collect information, which also does not adequately account for the psychological characteristics of the participants, but using the NHANES database, there have been several studies of OSA [34]. Second, the associations varied by sex and the interpretation of these findings is unclear and requires further study. Third, We cannot establish causality on the basis of this cross-sectional study, and intervention studies are needed for further confirmation. In this study, the prevalence of OSA in Americans aged 18 years and older is 52.95%, which is much higher than the worldwide OSA prevalent [35]. Different screening tools for OSA may be the cause of this phenomenon, but it still underscores the importance of preventing OSA. Although this study did not establish a causal link between SB and OSA, its substantial contribution lies in affirming the association between SB and OSA. This lays a foundation for guiding future research in this area.

Sedentary behavior can be subdivided into several types. Leisure Sedentary Behavior(LSB) usually refers to sedentary television watching, computer use, etc., that is characterized by an energy expenditure of less than 1.5 metabolic equivalents in a sitting or reclinging position [36]. Work-related sedentary behavior may lead to increased morbidity and mortality of cardiovascular disease, obesity and other diseases [37]. Based on the mental activity involved in sedentary behavior, it can be divided into mental active sedentary behavior characterized by mental demands (office work, reading, problem solving) and mental passive sedentary behavior (watching television) [38]. Sedentary behavior may be associated with increased risk for OSA, but it is uncertain whether all types of sedentary behavior have equal adverse effects. Therefore, future research can further explore and improve the effects of different types of sedentary behaviors on the prevalence of OSA, so as to promote the development and progress in related fields.

## Conclusion

In conclusion, our study demonstrated a positive association of SB with OSA after adjustment for anthropometric and clinical potential confounders. Furthermore, the positive effect of SB on OSA was more significant in women. Because the cross-sectional study could not prove causality, to explore the mechanisms underlying the positive association between SB and OSA, further studies are needed.

### Electronic supplementary material

Below is the link to the electronic supplementary material.

Supplementary Material 1

Supplementary Material 2

## Data Availability

The study analysed publicly available data, which is available here: https://www.cdc.gov/nchs/nhanes/index.htm.

## References

[1] Lv R, Liu X, Zhang Y, Dong N, Wang X, He Y (2023). Pathophysiological mechanisms and therapeutic approaches in obstructive sleep apnea syndrome. Signal Transduct Target Therapy.

[2] Andrade AG, Bubu OM, Varga AW, Osorio RS (2018). The relationship between Obstructive Sleep Apnea and Alzheimer’s Disease. J Alzheimer’s Disease: JAD.

[3] Scarinci F, Patacchioli FR, Ghiciuc CM, Pasquali V, Bercea RM, Cozma S et al. Psychological Profile and distinct salivary cortisol Awake Response (CAR) in two different study populations with obstructive sleep apnea (OSA) and Central Serous Chorioretinopathy (CSC). J Clin Med. 2020;9(8). 10.3390/jcm9082490 PMC7464438 32756367

[4] Benjafield AV, Ayas NT, Eastwood PR, Heinzer R, Ip MSM, Morrell MJ (2019). Estimation of the global prevalence and burden of obstructive sleep apnoea: a literature-based analysis. Lancet Respiratory Med.

[5] Jaiswal SJ, Owens RL, Malhotra A (2017). Raising awareness about sleep disorders. Lung India: Official Organ Indian Chest Soc.

[6] Watson NF (2016). Health Care savings: the Economic Value of Diagnostic and Therapeutic Care for Obstructive Sleep Apnea. J Clin Sleep Medicine: JCSM : Official Publication Am Acad Sleep Med.

[7] Surani S, Taweesedt P. Obstructive Sleep Apnea: New Perspective. Medicina (Kaunas, Lithuania). 2022;59(1). 10.3390/medicina59010075 PMC9862185 36676699

[8] Li MX, Wang Y, Hua SC, Li CM, Wang MP, Liu Y (2005). [The prevalence of obstructive sleep apnea-hypopnea syndrome in adults aged over 20 years in Changchun city]. Zhonghua Jie he he Hu Xi Za Zhi = Zhonghua Jiehe he huxi zazhi = Chinese. J Tuberculosis Respiratory Dis.

[9] Malhotra A, Orr JE, Owens RL (2015). On the cutting edge of obstructive sleep apnoea: where next?. Lancet Respiratory Med.

[10] Owen N, Healy GN, Matthews CE, Dunstan DW (2010). Too much sitting: the population health science of sedentary behavior. Exerc Sport Sci Rev.

[11] Young DR, Hivert MF, Alhassan S, Camhi SM, Ferguson JF, Katzmarzyk PT (2016). Sedentary Behavior and Cardiovascular Morbidity and Mortality: A Science Advisory from the American Heart Association. Circulation.

[12] Buman MP, Kline CE, Youngstedt SD, Phillips B, Tulio de Mello M, Hirshkowitz M (2015). Sitting and television viewing: novel risk factors for sleep disturbance and apnea risk? Results from the 2013 National Sleep Foundation Sleep in America Poll. Chest.

[13] Korshøj M, Banghøj AM, Winckler K, Sølund J, Tarnow L. Frequency of obstructive sleep apnoea in Danish truck drivers. Dan Med J. 2020;67(9). 32800071

[14] Liu Y, Yang L, Stampfer MJ, Redline S, Tworoger SS, Huang T. Physical activity, sedentary behaviour and incidence of obstructive sleep apnoea in three prospective US cohorts. Eur Respir J. 2022;59(2). 10.1183/13993003.00606-2021 PMC8933852 34289976

[15] Thosar SS, Bhide MC, Katlaps I, Bowles NP, Shea SA, McHill AW (2021). Shorter Sleep predicts longer subsequent day sedentary duration in healthy midlife adults, but not in those with Sleep Apnea. Nat Sci Sleep.

[16] You Y, Chen Y, Yin J, Zhang Z, Zhang K, Zhou J (2022). Relationship between leisure-time physical activity and depressive symptoms under different levels of dietary inflammatory index. Front Nutr.

[17] Cavallino V, Rankin E, Popescu A, Gopang M, Hale L, Meliker JR (2022). Antimony and sleep health outcomes: NHANES 2009–2016. Sleep Health.

[18] Ussery EN, Fulton JE, Galuska DA, Katzmarzyk PT, Carlson SA (2018). Joint prevalence of sitting time and leisure-time physical activity among US adults, 2015–2016. JAMA.

[19] Huang Y, Xu P, Fu X, Ren Z, Cheng J, Lin Z (2021). The effect of triglycerides in the associations between physical activity, sedentary behavior and depression: an interaction and mediation analysis. J Affect Disord.

[20] Booth HP, Charlton J, Gulliford MC (2017). Socioeconomic inequality in morbid obesity with body mass index more than 40 kg/m(2) in the United States and England. SSM - Popul Health.

[21] Flegal KM, Kruszon-Moran D, Carroll MD, Fryar CD, Ogden CL (2016). Trends in obesity among adults in the United States, 2005 to 2014. JAMA.

[22] You Y, Chen Y, Fang W, Li X, Wang R, Liu J (2022). The association between sedentary behavior, exercise, and sleep disturbance: a mediation analysis of inflammatory biomarkers. Front Immunol.

[23] Pratt M (2021). What’s new in the 2020 World Health Organization guidelines on physical activity and sedentary behavior?. J Sport Health Sci.

[24] Cao C, Friedenreich CM, Yang L (2022). Association of Daily Sitting Time and leisure-time physical activity with Survival among US Cancer survivors. JAMA Oncol.

[25] Pitta RM, Cerazi BG, Queiroga L, Ritti Dias RM, Mello MT, Cesena FHY (2022). Are physical inactivity, sitting time and screen time associated with obstructive sleep apnea in adults? A cross-sectional study. Sao Paulo Med J = Revista paulista de Med.

[26] White LH, Bradley TD (2013). Role of nocturnal rostral fluid shift in the pathogenesis of obstructive and central sleep apnoea. J Physiol.

[27] Elias RM, Bradley TD, Kasai T, Motwani SS, Chan CT (2012). Rostral overnight fluid shift in end-stage renal disease: relationship with obstructive sleep apnea. Nephrology, dialysis, transplantation: official publication of the European Dialysis and Transplant Association. Eur Ren Association.

[28] Ravesloot MJ, van Maanen JP, Dun L, de Vries N (2013). The undervalued potential of positional therapy in position-dependent snoring and obstructive sleep apnea-a review of the literature. Sleep Breath = Schlaf Atmung.

[29] Mirrakhimov AE (2013). Physical exercise related improvement in obstructive sleep apnea. Look for the rostral fluid shift. Med Hypotheses.

[30] Mendelson M, Lyons OD, Yadollahi A, Inami T, Oh P, Bradley TD (2016). Effects of exercise training on sleep apnoea in patients with coronary artery disease: a randomised trial. Eur Respir J.

[31] Redolfi S, Yumino D, Ruttanaumpawan P, Yau B, Su MC, Lam J (2009). Relationship between overnight rostral fluid shift and obstructive sleep apnea in nonobese men. Am J Respir Crit Care Med.

[32] Heinzer R, Vat S, Marques-Vidal P, Marti-Soler H, Andries D, Tobback N (2015). Prevalence of sleep-disordered breathing in the general population: the HypnoLaus study. Lancet Respiratory Med.

[33] Senaratna CV, Perret JL, Lodge CJ, Lowe AJ, Campbell BE, Matheson MC (2017). Prevalence of obstructive sleep apnea in the general population: a systematic review. Sleep Med Rev.

[34] Zhu H, Wu M (2023). A cross-sectional study on the relationship between electronic cigarette and combustible cigarette use with obstructive sleep apnea among U.S. adults: result from NHANES 2015–2018. Archives Public Health = Archives belges de sante Publique.

[35] Lyons MM, Bhatt NY, Pack AI, Magalang UJ (2020). Global burden of sleep-disordered breathing and its implications. Respirol (Carlton Vic).

[36] Chen X, Hong X, Gao W, Luo S, Cai J, Liu G (2022). Causal relationship between physical activity, leisure sedentary behaviors and COVID-19 risk: a mendelian randomization study. J Translational Med.

[37] van Uffelen JG, Wong J, Chau JY, van der Ploeg HP, Riphagen I, Gilson ND (2010). Occupational sitting and health risks: a systematic review. Am J Prev Med.

[38] Hallgren M, Vancampfort D, Owen N, Rossell S, Dunstan DW, Bellocco R (2020). Prospective relationships of mentally passive sedentary behaviors with depression: mediation by sleep problems. J Affect Disord.

