# Supplementary material for: The association between sedentary behavior and obstructive sleep apnea: a cross-sectional study from the NHANES (2007–2008 to 2015–2020)
Source: BMC Oral Health. 2024 Feb 12;24:224. doi: 10.1186/s12903-024-03960-0 (PMC10863124; doi:10.1186/s12903-024-03960-0)
Supplement: Supplementary file 1 — Supplementary Material 1 [file 12903_2024_3960_MOESM1_ESM.docx]

| **Table S1.** Baseline Participant Characteristics(N=10,651) | | | |
| --- | --- | --- | --- |
|  | Obstructive Sleep Apnea(OSA) | |  |
| Characteristics | Men | Women | P-value |
| **Age** |  |  | 0.159 |
| ＜44 | 2049 (36.17%) | 1716 (34.42%) | |
| [44,60) | 1627 (28.72%) | 1458 (29.24%) | |
| ≥60 | 1989 (35.11%) | 1812 (36.34%) | |
| **Race/Ethnicity** |  |  | <0.001 |
| Non–Hispanic white | 2169 (38.29%) | 1758 (35.26%) | |
| Non–Hispanic black | 1198 (21.15%) | 1252 (25.11%) | |
| Mexican American | 905 (15.98%) | 771 (15.46%) | |
| Other Race/Ethnicity | 1393 (24.59%) | 1205 (24.17%) | |
| **Education Level** |  |  | 0.093 |
| Below high school | 1422 (25.10%) | 1167 (23.41%) | |
| High school | 1353 (23.88%) | 1159 (23.25%) | |
| College or above | 2725 (48.10%) | 2512 (50.38%) | |
| **Marital Status** |  |  | <0.001 |
| Never married | 761 (13.43%) | 734 (14.72%) | |
| Married/Living with partner | 3982 (70.29%) | 2627 (52.69%) | |
| Widowed/Divorced/Separated | 761 (13.43%) | 1474 (29.56%) | |
| **PIR** |  |  | <0.001 |
| ＜1 | 896 (15.82%) | 1023 (20.52%) | |
| [1, 3) | 2205 (38.92%) | 1956 (39.23%) | |
| ≥3 | 1919 (33.87%) | 1415 (28.38%) | |
| **Alcohol Drinkers** |  |  | <0.001 |
| Non-drinkers | 353 (6.23%) | 755 (15.14%) | |
| Moderate alcohol use | 2268 (40.04%) | 1895 (38.01%) | |
| Heavy alcohol use | 2359 (41.64%) | 1675 (33.59%) | |
| **BMI** |  |  | <0.001 |
| ＜25 | 1069 (18.87%) | 861 (17.27%) | |
| [25,30) | 1888 (33.33%) | 1281 (25.69%) | |
| [30,40) | 1945 (34.33%) | 1801 (36.12%) | |
| ≥40 | 409 (7.22%) | 709 (14.22%) | |
| **HBP** |  |  | 0.106 |
| No | 1918 (33.86%) | 1740 (34.90%) | |
| Yes | 3409 (60.18%) | 2912 (58.40%) | |
| **Diabetes** |  |  | <0.001 |
| No | 376 (6.64%) | 483 (9.69%) | |
| Yes | 985 (17.39%) | 933 (18.71%) | |
| **PA** |  |  | <0.001 |
| Light | 1059 (18.69%) | 966 (19.37%) | |
| Moderate to vigorous | 1537 (27.13%) | 930 (18.65%) | |
| **SB** |  |  | 0.196 |
| ＜4h/d | 1714 (30.26%) | 1424 (28.56%) | |
| 4 to＜6h/d | 1399 (24.70%) | 1245 (24.97%) | |
| 6 to 8h/d | 1497 (26.43%) | 1329 (26.65%) | |
| ＞8h/d | 1055 (18.62%) | 988 (19.82%) | |
| **Sleep duration** |  |  | <0.001 |
| ＜7h | 1909 (33.70%) | 1468 (29.44%) | |
| [7,9] | 3312 (58.46%) | 2939 (58.95%) | |
| ＞9h | 410 (7.24%) | 557 (11.17%) | |
| **Smokers** |  |  | <0.001 |
| Never smoker | 2511 (44.32%) | 3089 (61.95%) | |
| Former smoker | 1760 (31.07%) | 976 (19.57%) | |
| Current smoker | 1334 (23.55%) | 877 (17.59%) | |
| Results in the table: N(%)  Numbers that do not add up to 100% are attributable to missing data. | | | |
