# Supplementary material for: The association between sedentary behavior and obstructive sleep apnea: a cross-sectional study from the NHANES (2007–2008 to 2015–2020)
Source: BMC Oral Health. 2024 Feb 12;24:224. doi: 10.1186/s12903-024-03960-0 (PMC10863124; doi:10.1186/s12903-024-03960-0)
Supplement: Supplementary file 2 — Supplementary Material 2 [file 12903_2024_3960_MOESM2_ESM.docx]

| **Table 2.** The association between Sedentary Behavior and OSA. | | | |
| --- | --- | --- | --- |
|  | Model Ⅰ  OR(95%CI)*p*-value | Model Ⅱ  OR(95%CI)*p*-value | Model Ⅲ  OR(95%CI)*p*-value |
| Sedentary Behavior（hours/day） | |  |  |
| 0 to<4 | reference | reference | reference |
| 4 to<6 | 1.10 (0.96, 1.26) 0.162 | 1.12 (0.98, 1.28) 0.112 | 1.13 (0.98, 1.30) 0.085 |
| 6 to 8 | 1.22 (1.08, 1.39) 0.002 | 1.24 (1.09, 1.42) 0.001 | 1.25 (1.09, 1.44) 0.001 |
| >8 | 1.26 (1.09, 1.45) 0.002 | 1.28 (1.11, 1.48) 0.001 | 1.28 (1.10, 1.49) 0.001 |
| *P* for trend | <0.001 | <0.001 | <0.001 |
| OR, odds ratio; CI, confidence intervals; OSA, Obstructive sleep apnea. | | | |
